# Intravenous administration of anti-vascular endothelial growth factor humanized monoclonal antibody bevacizumab improves articular cartilage repair

**DOI:** 10.1186/ar3142

**Published:** 2010-09-24

**Authors:** Toshihiro Nagai, Masato Sato, Toshiharu Kutsuna, Mami Kokubo, Goro Ebihara, Naoshi Ohta, Joji Mochida

**Affiliations:** 1 Department of Orthopaedic Surgery, Surgical Science, Tokai University School of Medicine, 143 Shimokasuya, Isehara, Kanagawa 259-1193, Japan

## Abstract

**Introduction:**

In this study, we investigate the efficacy of repairing an osteochondral defect in rabbit knee joints by administering bevacizumab, a humanized monoclonal anti-vascular endothelial growth factor (VEGF) antibody.

**Methods:**

An osteochondral defect was created on the patellar groove of 20 Japanese white rabbits that were classified into two recipient groups: group B, administration of bevacizumab (100-mg intravenous injection on the day of surgery and 2 weeks later), and a control group (defect only). Rabbits were killed 1 and 3 months postoperatively. Sections were stained with safranin O. Repair sites were evaluated using the modified O'Driscoll International Cartilage Repair Society grading system. The expression of chondromodulin (ChM)-I and VEGF was evaluated using immunohistochemical analyses.

**Results:**

At 1 month postoperatively, the repair site in group B was filled with cartilaginous tissue. At 3 months, the repair site retained this cartilage phenotype. At 1 month in the controls, the defects were mainly filled with fibrous tissue. At 3 months, the defect was replaced by fibrous tissue and bone. Over the 3-month period, histological scores were significantly higher in group B than in the controls. At 1 month, group B showed intense positive results for ChM-I in the bottom of the repair tissue. VEGF was also identified in the same area. In the controls, no ChM-I was observed in the repair tissue. Conversely, the remodeling hypertrophic chondrocyte layer stained intensely for VEGF.

**Conclusions:**

Intravenous administration of bevacizumab contributes to better repair of articular cartilage in an osteochondral defect model. We suggest the possibility of facilitating articular cartilage repair with anti-VEGF antibody rather than using cultured cells or artificial scaffolds.

## Introduction

Mature articular cartilage shows limited capacity for regeneration after degeneration or injury [1]. For this reason, various treatments have been developed in anticipation of restoration by regenerative medicine. At present, techniques using penetration of subchondral bone [2-5], microfracture [6-9], mosaicplasty [10-12], cell transplantation [13-16], and implantation of tissue-engineered cartilage with various scaffold materials [17-22] or without scaffold [23-27] have been developed to overcome this obstacle. Penetration of subchondral bone such as drilling and microfracture to be filled with reparative cells from bone marrow is a method that has been developed to stimulate spontaneous healing [18]. This procedure attempts to achieve repair via the mechanism of endochondral ossification. However, the defect to be filled with reparative cells shows a large amount of vascular invasion, and the tissue tends to be replaced by bone and a surface of fibrocartilaginous repair tissue [28].

Successful regeneration of any tissue requires the presence of reparative cells with the potential to differentiate into the phenotypes required to restore the damaged site, but a microenvironment that supports the proliferation and differentiation of those cells is also needed [28,29]. In anticipation of favorable articular cartilage repair in the osteochondral defect model, reparative cells must be provided with an environment to acquire the properties of natural articular cartilage. We recently constructed a 3-D, scaffold-free, tissue-engineered cartilage [24] and transplanted this cartilage in only the superficial layer region of the osteochondral defects as an initiator of cartilage differentiation in reparative cells [23] and achieved good restoration effects in the long term [29]. We confirmed that in the early stage of transplantation, a good restoration effect of articular cartilage is seen with reparative cells derived from marrow that acquire antiangiogenic properties [23]. We therefore hypothesized that good cartilage repair may be achieved by inhibiting the bioactivity of vascular endothelial growth factor (VEGF) in the osteochondral defect. A recent investigation examined the effect of treatment with anti-VEGF humanized monoclonal antibody (bevacizumab), which was developed as a treatment for malignant tumors [30]. Bevacizumab binds to VEGF secreted by angiogenic tumors and thereby inhibits VEGF binding to the VEGF receptor in vascular endothelial cells, reportedly restraining cancer growth by inhibiting angiogenesis [31,32].

The objective of this study is to investigate the efficacy of repair in an osteochondral defect model of the rabbit knee joint following administration of bevacizumab, a humanized monoclonal anti-VEGF antibody, without using cultured cells or artificial scaffolds.

## Materials and methods

Animal experiments were approved by the ethics review board of Tokai University and were performed in accordance with the guidelines on animal use of Tokai University.

### Repair of the osteochondral defect

Twenty Japanese white rabbits (female, 16-18 weeks old, weighing approximately 3 kg) were used in this study. The rabbits were anesthetized by exposure to sevoflurane and O_2 _gas. After receiving a medial parapatellar incision to both legs, each patella was dislocated laterally and an osteochondral defect (diameter, 5 mm; depth, 3 mm) was created on the patellar groove of the femur in both legs using a drill and a biopsy punch (Kai Industries, Seki, Japan). The bottom of the subchondral bone was shaved to a plane using the biopsy punch until bleeding was seen from the marrow. Rabbits were classified into two recipient groups: Group B, with administration of bevacizumab (10 rabbits; 100-mg intravenous injection administered on the day of surgery and 2 weeks later); and controls (10 rabbits; defect only). After recovery from surgery, all animals were allowed to walk freely in their cages without any splints.

### Histological evaluation of cartilage repair

Rabbits were killed 1 and 3 months postoperatively by an overdose of intravenous anesthetic. The distal part of the femur was excised and fixed with 4% paraformaldehyde for 7 days. Each specimen was decalcified in a solution of 10% ethylenediaminetetraacetic acid (EDTA) in distilled water (pH 7.4) for 2-3 weeks, then embedded in paraffin wax and sectioned perpendicularly (4.5-m sections) through the center of the defect. Each section was stained with safranin O for glycosaminoglycans.

Immunohistochemistry was performed as described previously [23,33]. Briefly, sections were deparaffinized according to standard procedures. Sections were treated with 0.005% proteinase (type XXIV; Sigma-Aldrich Co., St. Louis, MO, USA) for 30 min at 37°C for antigen retrieval. For chondromodulin-I (ChM-I), primary goat polyclonal antibody (Santa Cruz Biotechnology, Santa Cruz, CA, USA) diluted 1:200 in phosphate-buffered saline (PBS) and 1% bovine serum albumin (BSA) was placed on the section overnight at 4°C. For VEGF, a primary mouse monoclonal antibody (Upstate, Lake Placid, NY, USA) diluted 1:50 in PBS and 1% BSA was placed on the section overnight at 4°C. Slides were washed with PBS after incubation for 1 h at room temperature with biotin-conjugated goat antimouse secondary antibody for VEGF and with biotin-conjugated donkey antigoat secondary antibody for ChM-I. Next, slides were treated with horseradish peroxidase-labeled streptavidin for 1 h and then soaked in 0.05% solution of diaminobenzidine in Tris HCl buffer (pH 7.6) containing 0.005% hydrogen peroxide. Finally, slides were counterstained with Mayer's hematoxylin. Safranin O-stained sections were scored by two individuals under blinded conditions, according to a modified O'Driscoll [34] International Cartilage Repair Society (ICRS) grading scale [26] (Table 1).

**Table 1:** Histological grading system^a^

Tissue morphology (Ti)	Intactness of calcified cartilage layer, formation of tidemark (Tide)	Lateral integration of implanted material (Latl)
4 = Mostly hyaline cartilage	1 = <25% of the calcified cartilage layer intact	1 = Not bonded
3 = Mostly fibrocartilage	2 = 25-49% of the calcified cartilage layer intact	2 = Bonded at one end/partially both ends
2 = Mostly noncartilage	3 = 50-75% of the calcified cartilage layer intact	3 = Bonded at both sides
1 = Exclusively noncartilage	4 = 76-90% of the calcified cartilage layer intact	**Basal integration of implanted material (Basl)**
**Matrix staining (Matx)**	5 = Complete intactness of the calcified cartilage layer	1 = <50%
1 = None	**Subchondral bone formation (Bform)**	2 = 50-70%
2 = Slight	1 = No formation	3 = 70-90%
3 = Moderate	2 = Slight	4 = 91-100%
4 = Strong	3 = Strong	**Inflammation (InfH)**
**Structural integrity (Stru)**	**Histologic appraisal of surface architecture (SurfH)**	1 = Strong inflammation
1 = Severe disintegration	1 = Severe fibrillation	3 = Slight inflammation
2 = Cysts or disruption	2 = Moderate fibrillation	5 = No inflammation
3 = No organization of chondrocytes	3 = Slight fibrillation or irregularity	**Histologic grading system (Hgtot)**
4 = Beginning of columnar organization of chondrocytes	4 = Normal	Some of the histologic variables:
5 = Normal, similar to healthy mature cartilage	**Histologic appraisal defect filling (FilH)**	Tissue morphology (Ti)
**Chondrocytes clustering in implant (Clus)**	1 = <25%	Matrix staining (Matx)
1 = 25-100% of cells clustered	2 = 26-50%	Structural integrity (Stru)
2 = <25% of the cells clustered	3 = 51-75%	Cluster formation (Clus)
3 = No clusters	4 = 76-90%	Tidemark opening (Tide)
	5 = 91-110%	Bone formation (Bform)
		Histologic surface architecture (SurfH)
		Histologic degree of defect filling (FilH)
		Lateral integration of defect-filling tissue (Basl) and histologic signs of inflammation (InfH)

^a^Presentation of variables and the grading system based on a modified International Cartilage Repair Society (ICRS) grading scale [26] developed by O'Driscoll, Keeley and Salter [34].

### Statistical analysis

Results are presented as the means ± standard deviation (SD). Histological score was analyzed by the Mann-Whitney *U *test. Values of *P *< 0.05 were considered statistically significant for any differences.

## Results

### Histological evaluation of repair tissue

Operations were uneventful, and all rabbits immediately resumed normal cage activity. In Group B, major infection was identified in one knee at 1 month after surgery and in three knees at 3 months. These infected knees were omitted from the study. As a result, nine knees at 1 month and seven knees at 3 months were assessed from Group B, compared to 10 knees in controls at both 1 and 3 months.

**Figure 1:**
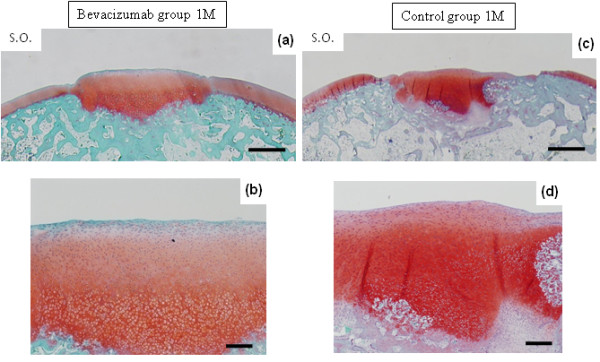
**Photomicrographs showing safranin O staining at 1 month after surgery**. **(a, b) **The defect treated with bevacizumab. **(c, d) **The defect untreated as the control. Scale bars, 1 mm in (a) and (c) and 250 μm in (b) and (d).

**Figure 2:**
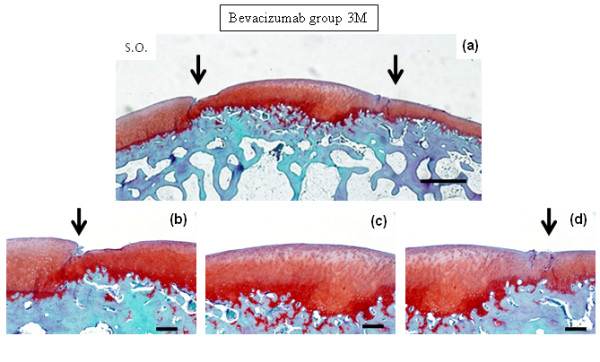
**Photomicrographs show safranin O staining at 3 months after surgery**. The defect was treated with bevacizumab. Bars, 1 mm in **(a) **and 250 μm in **(b, c **and **d)**.

**Figure 3:**
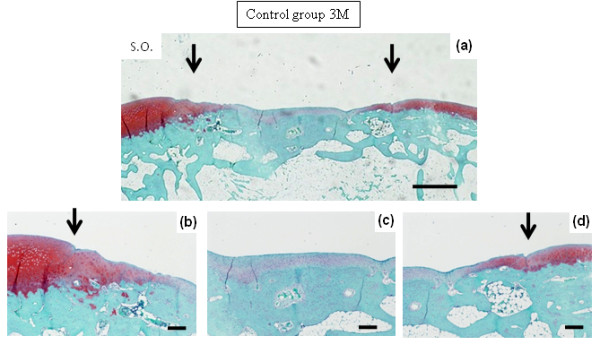
**Photomicrographs show safranin O staining at 3 months after surgery**. The untreated defect in the control. Bars, 1 mm in **(a) **and 250 μm in **(b, c **and **d)**.

At 1 month after surgery, defects in both Group B and the controls were filled with reparative cells. In Group B, the repair site appeared to be filled with cartilaginous tissue, which was stained with safranin O (Figures 1a and 1b). Lateral integration was well bonded at both sides of the surrounding cartilage. The surface of the repair site showed several smooth fibrous cell layers, and rounded chondrocytes formed inside the repair tissue in a convex pattern. The lower portion of the repair tissue contained hypertrophic chondrocytes that were remodeling the subchondral bone. Thus, the defects showed sequential construction of abundant inhomogeneous extracellular matrix from the surface by fibrous cells (or fibrocartilage cells), rounded chondrocytes and hypertrophic chondrocytes. Similarly, in the controls, defects consisted of inhomogeneous extracellular matrix from fibrous cells (or fibrocartilage cells), rounded chondrocytes and hypertrophic chondrocytes; however, no tendency toward uniform constitution was apparent. The defect was not filled with repaired tissue (Figures 1c and 1d). At 3 months in Group B, the repair site maintained a cartilage phenotype and was well integrated with the surrounding cartilage (Figure 2). Repaired tissue showed a columnar organization. The surface of the repair site retained a smooth and convex formation similar to the surrounding cartilage. In the controls at 3 months, the repair site had been replaced with fibrous tissue and bone (Figure 3), and in the surrounding adjacent cartilage there was evidence of osteoarthritic changes with loss of cartilage.

### Evaluation of ChM-I and VEGF expression

**Figure 4:**
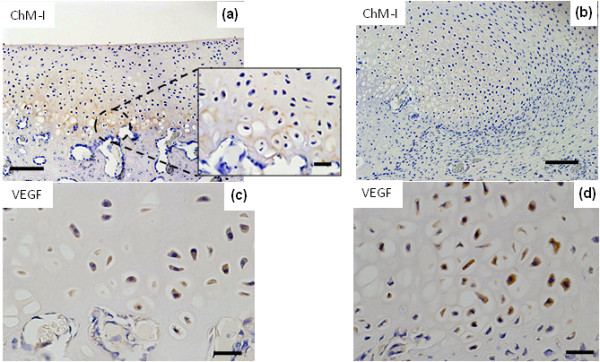
**Chondromodulin (ChM)-I and vascular endothelial growth factor (VEGF) localized in cartilage at 1 month after surgery**. **(a, c) **The defect treated with bevacizumab. **(b, d) **The defect in the control. (a) In the bevacizumab group, ChM-I immunostaining is detected in the interterritorial matrix of the remodeling site. (b) In the control group, ChM-I immunostaining is not detected in the remodeling site. (c, d) In both groups, VEGF immunostaining is detected in the remodeling site of the reparative cells. Bars, 100 μm in (a) and (b) and 25 μm in (c) and (d).

At 1 month, Group B showed intense positive results for ChM-I at the bottom of the repair tissue in the remodeling hypertrophic chondrocyte layer, representing the border between cartilage and bone (Figure 4a). ChM-I had accumulated in the interterritorial space of the repaired matrix (Figure 4a). In the controls, no ChM-I was observed in the repair tissue (Figure 4b). Conversely, the remodeling hypertrophic chondrocyte layer was intensely positive for VEGF in both Group B and in the controls (Figures 4c and 4d).

### Histological scoring of repair tissue

We evaluated the repair site using a modified version of the grading system developed by O'Driscoll, Keeley and Salter [34]. In this system, 11 histologic categories were evaluated and scored: tissue morphology (Ti), matrix staining (Matx), structural integrity (Stru), cluster formation (Clus), tidemark opening (Tide), bone formation (Bform), histologic appraisal of surface architecture (SurfH), histologic appraisal of the degree of defect filling (FilH), lateral integration of defect-filling tissue (Latl), basal integration of defect-filling tissue (Basl) and histologic signs of inflammation (InfH). The total score ranged from 11 (no repair) to 45 (normal articular cartilage) (Table 1).

At 1 month, inside the repair tissue in Group B, Ti was mostly hyaline cartilage in seven of nine cases, with high cellularity of rounded chondrocytes and mostly fibrocartilage in two of nine cases. Conversely, in the controls, 4 of 10 cases showed mostly hyaline cartilage, 4 of 10 cases were mostly fibrocartilage and 2 of 10 cases were mostly noncartilage. For Matx in Group B, six of nine cases were strong, two of nine cases were moderate and one case showed slight staining. In the controls, 5 of 10 cases were strong, 3 of 10 cases were moderate and 2 of 10 cases showed slight staining. In Group B, Stru of the defect filling revealed the beginning of columnar organization of chondrocytes in six of nine cases and no organization of chondrocytes in three of nine cases. In the controls, 3 of 10 cases showed the beginning of columnar organization, 4 of 10 cases showed no organization and 3 of 10 cases showed cysts or disruptions. In both groups, Clus was not observed except in one instance. In one instance, there was a small amount of Clus in both groups. Also, in both groups, Tide was opened in all instances. In Group B, subchondral Bform was not recognized in four of nine cases, slightly recognized in four of nine cases and strongly recognized in one of nine cases. In the control group, subchondral Bform was not recognized in 8 of 10 cases and was slightly recognized in 2 of 10 cases. SurfH in Group B was normal in five of nine cases and showed slight fibrillation or irregularity in four of nine cases; in the control group, 1 of 10 cases was normal, 6 of 10 cases showed slight fibrillation or irregularity, 2 of 10 cases showed moderate fibrillation or irregularity and 1 of 10 cases showed severe fibrillation or disruption. FilH in Group B was complete in six of nine cases and nearly complete in three of nine cases. In controls, FilH was complete in 2 of 10 cases, nearly complete in 4 of 10 cases, moderate in 2 of 10 cases and nearly empty in 2 of 10 cases. Latl in Group B was bonded on both sides in seven of nine cases and bonded at one end or partially at both ends in two of nine cases. In controls, Latl was bonded on both sides in 1 of 10 cases, bonded at one end or partially at both ends in 6 of 10 cases and not bonded in 3 of 10 cases. Basl was good in all cases for both groups. In Group B, no inflammation was observed in all cases for InfH. The control group showed no inflammation in 5 of 10 cases, slight inflammation in 4 of 10 cases and strong inflammation in 1 of 10 cases. As a result, at 1 month, the total score was significantly higher for Group B than for the controls. In terms of individual scores, SurfH, FilH and Latl for Group B were significantly higher than in controls.

In Group B, Ti at 3 months showed mostly hyaline cartilage in six of seven cases. In one of seven cases, tissue was mostly fibrocartilage. Conversely, in the controls, 3 of 10 cases were mostly hyaline cartilage, with mostly fibrocartilage in 1 of 10 cases and exclusively noncartilage in 6 of 10 cases. For Matx, six of seven cases in Group B showed strong staining and one of seven cases showed moderate staining; in the controls, 3 of 10 cases were strong, one instance was moderate and 6 of 10 cases were nonstaining. In Group B, Stru of the defect filling revealed tissue similar to healthy mature cartilage in three of seven cases, beginning columnar organization of chondrocytes in three of seven cases and no organization of the chondrocytes in one of seven cases. In the controls, only one instance was similar to healthy mature cartilage, 2 of 10 cases were beginning columnar organization, 1 of 10 cases had no organization and 6 of 10 cases showed severe disintegration. In Group B, Clus was not observed. In controls, 2 of 10 cases showed no clusters, 2 of 10 cases showed some clusters and 6 of 10 cases showed abundant cluster cells or nonchondrocytes. Tide in Group B was complete in three of seven cases, nearly complete in two of seven cases, half degree in one of seven cases and nearly absent in one of seven cases; in the controls, 1 of 10 cases was complete, 2 of 10 cases were nearly complete, 1 of 10 cases was half degree and 6 of 10 cases were not recognized as containing a calcified cartilage layer. Bform was recognized in both groups, except for one instance in each. In one instance, there was a slightly Bform in both groups. SurfH in Group B was normal in five of seven cases and showed slight fibrillation or irregularity in two of seven cases; in the controls, 1 of 10 cases was normal, 1 of 10 cases showed slight fibrillation or irregularity, 2 of 10 cases showed moderate fibrillation or irregularity and 6 of 10 cases showed severe fibrillation or disruption. FilH in Group B was complete in all instances. In the controls, FilH was complete in 4 of 10 cases, moderate in 1 of 10 cases, nearly empty in 1 of 10 cases and almost empty in 4 of 10 cases. Latl in Group B were bonded at both sides in six of seven cases and bonded at one end in one of seven cases. In the controls, Latl was bonded both sides in 1 of 10 cases, bonded at one end or partially both ends in 2 of 10 cases and not bonded in 6 of 10 cases. Basl was good in all cases in both groups. For InfH, no inflammation was observed in any cases in either group. At 3 months, the total score for Group B was significantly higher than that for the controls. In terms of individual scores, Ti, Matx, Stru, Clus, SurfH, FilH and Latl were significantly higher for Group B than for the control group (Table 2).

**Table 2:** Histological scores for cartilage repair at 1 month and 3 months after surgery^a^

	1 month		3 months	
		
	Group B	Control	Group B	Control
Ti	3.77 ± 0.4	3.20 ± 0.7	3.85 ± 0.3*	2.10 ± 1.4
Matx	3.55 ± 0.7	3.30 ± 0.8	3.71 ± 0.7*	2.10 ± 1.4
Stru	3.66 ± 0.5	3.00 ± 0.8	4.28 ± 0.7*	2.41 ± 1.6
Clus	2.88 ± 0.3	2.90 ± 0.3	3.00 ± 0.0*	1.60 ± 0.8
Tide	1.00 ± 0.0	1.00 ± 0.0	3.85 ± 1.4	2.20 ± 1.6
Bform	1.66 ± 0.7	1.20 ± 0.4	2.85 ± 0.3	2.90 ± 0.3
SurfH	3.55 ± 0.5*	2.70 ± 0.8	3.71 ± 0.4*	1.70 ± 1.0
FilH	4.66 ± 0.5*	3.70 ± 1.0	5.00 ± 0.0*	2.90 ± 1.9
Latl	2.77 ± 0.4*	1.80 ± 0.6	2.85 ± 0.3*	1.60 ± 0.8
Basl	4.00 ± 0.0	4.00 ± 0.0	4.00 ± 0.0	4.00 ± 0.0
InfH	5.00 ± 0.0	3.80 ± 1.3	5.00 ± 0.0	5.00 ± 0.0
Hgtot	36.55 ± 2.1*	30.50 ± 4.1	40.14 ± 2.5*	27.7 ± 10.0

^a^Values are means ± SD. The total score range is from 11 (no repair) to 45 (normal articular cartilage).

*Denotes significance at *P *< 0.05.

## Discussion

VEGF is overexpressed in numerous solid angiogenic tumors and hematological malignancies. Interrupting the VEGF pathway has thus become a major focus of oncology research [35]. The first approved antiangiogenic therapy was bevacizumab, a humanized monoclonal anti-VEGF antibody. Following the success of a pivotal trial, the FDA approved bevacizumab for use in combination with intravenous 5-fluorouracil-based chemotherapy as a treatment for patients with first-line or previously untreated metastatic cancer of the colon or rectum [36]. Bevacizumab is anticipated to be useful not only for cancer treatment but also as a major advance in antiangiogenic therapy.

Generally, osteochondral defects have access to reparative cells of the bone marrow [37]. This connection allows infiltration of the bone by mesenchymal stem cells (MSCs) from the bone marrow, which can then proliferate and differentiate. MSC-derived chondrocytes subsequently appear during endochondral ossification and are invaded by the vasculature and marrow, and eventually the defects are replaced by subchondral bone [28,38]. In summary, MSC-derived chondrocytes are spontaneously recruited as reparative cells in osteochondral defects and are replaced by bone with high levels of vascular invasion. However, articular cartilage is a naturally avascular tissue, except during skeletal development, when endochondral bone formation occurs. We speculate that MSCs may use different courses of differentiation to become bone or cartilage, based on environmental differences in vascularization and avascularization. We recently reported that MSCs that acquire antiangiogenic properties achieve good cartilage restoration [23]. Furthermore, previous research has shown that VEGF expression by chondrocytes in osteoarthritic joints may be related to articular cartilage destruction [39-46]. High-dose VEGF may induce the onset and progression of arthritis [47,48]. Also, expression of high levels of VEGF during the terminal stages of chondrogenesis leads to endochondral ossification through angiogenesis [49,50]. We therefore studied the restoration of articular cartilage by blocking VEGF signaling with bevacizumab in a model of osteochondral defects in Japanese white rabbits.

At 1 month after surgery in both Group B and the controls, defects were recruited with reparative cells from the marrow and synovial tissue, and this was composed of differentiated chondrocytes, hyperchondrocytes and fibrous cells. These heterogeneous cells stained positive for safranin O and showed various levels of structured organization. From the surface, these structures consisted of a fibrous or fibrocartilage layer, a hyaline cartilage layer and a hypertrophic chondrocyte layer in both groups. As a result, no significant differences were apparent between the two groups at 1 month postoperatively in Ti, Matx or Stru. However, at 1 month after surgery, the defects were repaired by hyaline cartilage (seven of nine cases) in Group B, a result of blocking VEGF, whereas in the controls, the defects were repaired with that of various tissues, including hyaline cartilage (4 of 10 cases), fibrocartilage (4 of 10 cases) and noncartilage (2 of 10 cases). There was no delay in subchondral bone formation in Group B when blocking VEGF. For autologous reparative cells, basal integration was good and most inflammatory signs were absent from both groups. On the other hand, FilH, Latl and SurfH were significantly higher for Group B than for the controls. Actually, at 1 month after surgery, six of nine cases in Group B showed convex surfaces of these repaired tissues in surrounding articular cartilage, compared to only 2 of 10 cases in the controls. These results indicate that blocking VEGF preserves the accumulation of reparative cells in the defect. This is supported by studies showing that VEGF treatment prevents condensation of chondrogenic mesenchyme during early limb bud development through abnormal vascularization [51]. As a sufficient number of reparative cells made contact with the surrounding cartilage layer, lateral integration was considered to be good. Also, repaired tissue taking a convex form consisted of a smoother surface than repaired tissue with a concave form.

Defects had been repaired by the formation of various tissues in the controls at 3 months after surgery, which included hyaline cartilage (3 of 10 cases), fibrocartilage (1 of 10 cases) and exclusively noncartilage (6 of 10 cases). On the other hand, when blocking VEGF, defects were repaired mostly with hyaline cartilage (six of seven cases), with only one case being mostly fibrocartilage. To emphasize this, no cases showed replacement by fibrous tissue or bone. Similarly, 1 month after surgery, the controls showed repair without consistent tissue morphology, while Group B showed repair with consistency of tissue morphology. At 3 months postoperatively, Ti, Matx, Stru and Clus were significantly higher for Group B than for the controls. In both Group B and the controls, Tide was generally closed and bone formation was gradually observed. Continuous basal integration was also good and most signs of inflammation were not apparent in either group. Tissue that was repaired in the form of fibrous tissue and bone tended to show moderate or severe fibrillation of surface architecture and low defect filling. Therefore, SurfH and FilH were significantly higher for Group B than for the controls. As mentioned before, a sufficient number of reparative cells were in contact with the surrounding cartilage layer, and lateral integration was considered to be good. As a result, at 3 months, the total score was significantly higher for Group B than for the controls.

Interestingly, ChM-I was expressed in the early stage of tissue repair after bevacizumab administration. ChM-I reportedly stimulates chondrocyte proliferation and proteoglycan synthesis in vitro and inhibits proliferation of vascular endothelial cells in vitro and in vivo [52,53]. Kitahara *et al*. [53] suggested that in their mouse model, ChM-I acts to inhibit vascular invasion in the immature state of articular cartilage, and levels of ChM-I gradually decrease with age thereafter. Hiraki *et al*. [52] reported that ChM-I is expressed in the avascular zone of cartilage in developing bone, but is not present in calcifying cartilage. Such findings suggest a regulatory role of ChM-I in vascular invasion during endochondral bone formation.

In this study, ChM-I was expressed at the bottom of the repair site invaded near the vasculature. ChM-I accumulated in the interterritorial space of the repaired matrix and was surrounding the cells that expressed VEGF. ChM-I is thought to form a barrier to inhibit vascular invasion from subchondral bone, indicating that it facilitates the acquisition of articular cartilage through the process of MSC differentiation in endochondral ossification. However, the shift from angiogenesis to antiangiogenesis is not determined entirely by ChM-I and VEGF. Inducers of endogenous angiogenic molecules also exist in the process of endochondral ossification, and these include VEGF [53], fibroblast growth factor 2 [54], transforming growth factor [55] and tissue matrix metalloprotease 9 [56].

In other studies, VEGF has been reported to be necessary for chondrocyte survival during cartilage development. In VEGF-deficient mouse models, massive cell death is observed in the joint and epiphyseal regions of cartilage during cartilage development [57,58]. In this study, we blocked VEGF temporarily to initiate reparative cells. To avoid complete inhibition of the bioactivity of VEGF in reparative cells, we blocked VEGF on the day of surgery and 2 weeks later. As a result, 1 month after surgery, reparative cells actually expressed VEGF in Group B (Figure 4c). Moreover, expression of ChM-I as an antiangiogenic factor was observed in the layer of reparative cells involved in blood vessel invasion from subchondral bone (Figure 4a). It is important to consider the efficacy of various VEGF treatments in the balance of angiogenesis-antiangiogenesis during chondrogenic differentiation of MSC-derived reparative cells. In other words, repair of articular cartilage may be achievable by adjusting optimal VEGF signaling. In a recent study, Kubo *et al*. [39] reported good cartilage restoration using muscle-derived stem cells that transfected with the genes of Flt-1 (a VEGF antagonist) and bone morphogenetic protein (BMP) 4 in a mouse osteochondral defect model. However, the techniques applied in that study were complicated, as they required isolation of stem cells from muscular tissue, gene transduction and cell culture in vitro, as well as cell transplantation. Conversely, the present technique involves the simple means of achieving cartilage restoration by intravenous administration of bevacizumab, a treatment already cleared for clinical application. Hence, this method would be applicable in many medical facilities. Thus, vascularization of the tissue environment after injury or degeneration would be improved to a more advantageous situation of cartilage repair if VEGF were blocked. Accordingly, without depending on cells or tissue transplantation, this approach would augment the curative effects of existing approaches such as microfracture or drilling.

The half-life of bevacizumab in the circulation of humans is reportedly 17-21 days. The approved dose of bevacizumab in humans is 5 mg/kg, and the clinical administration interval is more than 2 weeks [30]. Bevacizumab is cross-reactive with rabbit VEGF, but has eightfold lower affinity for rabbit VEGF than for human VEGF [59]. Therefore, in this study, we investigated bevacizumab at the dose of 40 mg/kg administered on the day of surgery and 2 weeks later. As a future consideration, we plan to investigate the dosage and duration of bevacizumab administration. We will also address the side effects of infection by applying minimally invasive surgery with an arthroscope and using antibiotics.

## Conclusions

Temporary intravenous administration of the humanized monoclonal anti-VEGF antibody bevacizumab in an osteochondral defect model results in positive restorative effects. We suggest that this approach would be useful to achieve repair of articular cartilage without the need for cells or tissue transplantation.

## Abbreviations

BASL: basal integration of defect-filling tissue; BFORM: bone formation; BMP: bone morphogenetic protein; BSA: bovine serum albumin; CHM-I: chondromodulin-I; CLUS: cluster formation; EDTA: ethylenediaminetetraacetic acid; FILH: histologic appraisal of the degree of defect filling; ICRS: International Cartilage Repair Society; INFH: histologic signs of inflammation; LATL: lateral integration of defect-filling tissue; MATX: matrix staining; PBS: phosphate-buffered saline; SD: standard deviation; STRU: structural integrity; SURFH: histologic appraisal of surface architecture; TI: tissue morphology; TIDE: tidemark opening; VEGF: vascular endothelial growth factor.

## Competing interests

The authors declare that they applied for a patent relating to the content of the manuscript in Japan, but did not receive any reimbursements, fees, funding or salary from an organization. The competitive companies developing or selling anti-VEGF drugs (e.g., Novartis, Wyeth, Bayer Schering) may keep them in check.

## Authors' contributions

TN and MS performed most of the experiments and MK performed the immunohistochemistry. TK, GE and NO helped with in vivo experiments. TN performed statistical analyses. MS and JM designed and coordinated the study and helped draft the manuscript. All authors approved the final manuscript.
